# Carotid ultrasound investigation as a prognostic tool for patients with diabetes mellitus

**DOI:** 10.1186/s12933-019-0895-0

**Published:** 2019-07-12

**Authors:** Matthias Hoke, Martin Schillinger, Erich Minar, Georg Goliasch, Christoph J. Binder, Florian J. Mayer

**Affiliations:** 1 Department of Internal Medicine II, Division of Angiology, Medical University of Vienna, Vienna, Austria; 2 Department of Internal Medicine II, Division of Cardiology, Medical University of Vienna, Vienna, Austria; 3 Department of Laboratory Medicine, Medical University of Vienna, Währinger Gürtel 18—20, 1090 Vienna, Austria

**Keywords:** Carotid atherosclerosis, Diabetes mellitus, Hba1c, Biomarker, Risk factor

## Abstract

**Background:**

Experimental and clinical data indicate a major influence of diabetes on atherogenesis. We aimed to assess whether the effect of diabetes on long-term mortality in asymptomatic patient with carotid stenosis is contingent upon the degree of the carotid atherosclerotic burden.

**Methods:**

1065 patients with neurological asymptomatic carotid atherosclerosis as evaluated by duplex sonography were prospectively followed for cause-specific mortality.

**Results:**

During a median of 11.8 years, a total of 549 deaths, including 362 cardiovascular deaths, were recorded. Diabetes and glycohemoglobin A1c (Hba1c) levels were significantly associated with mortality. Diabetes displayed an independent risk for all-cause (adjusted HR 1.62; 95% CI 1.35–1.94) and cardiovascular death (adjusted HR 1.75, 95% CI 1.40–2.19). The adjusted hazard ratio per increase of 1% of Hba1c levels was 1.21 (P < 0.01) for all-cause and 1.31 (P < 0.01) for cardiovascular mortality, respectively. Patients with diabetes mellitus and a higher degree of carotid stenosis and were at great risk of adverse outcome. Only 21% of the asymptomatic diabetic patients with carotid narrowing over 50% survived, whereas 62% of the patients without diabetes and with carotid atherosclerosis below 50% were still alive after 12-years of follow-up. The high risk for all-cause and cardiovascular death of these patients remained significant after adjustment for various established cardiovascular risk factors in multivariable regression analysis (adjusted hazard ratio 2.4, P < 0.001; compared to patients without diabetes and < 50% carotid atherosclerosis).

**Conclusion:**

Diabetic patients with carotid stenosis ≥ 50% are at exceptional high risk for all-cause and cardiovascular death. Thus, routinely ultrasound investigation of the carotid arteries might be a valuable prognostic tool for patients with diabetes mellitus.

## Background

Diabetes mellitus is associated with increased morbidity and mortality, and is linked to some acute but mainly chronic complications, based on functional and structural damages to the blood vessels. The disease is, unsurprisingly, a major risk factor for cardiovascular disease and a plethora of experimental and clinical studies link hyperglycemia to the development and progression of atherosclerosis. Various mechanisms by which diabetes contributes to cardiovascular disease and atherosclerosis have been identified. Alterations of the vessel wall, due to endothelial and smooth muscle cell dysfunction, are the main characteristics of diabetic vasculopathy. The chronic hyperglycemic state increases the generation of advanced glycation end products (AGEs), which are formed by sequential non-enzymatic reactions of glucose and other glycating compounds. When glucose mediates the reaction, initially the Amadori adduct fructosyl-lysine is formed [1]. In hemoglobin, this adduct is called glycohemoglobin A1c (HbA1c), which standardized glycemic control in diabetic patients. AGEs cause the development of reactive oxygen species, which leads to lipid peroxidation and generation of oxidized free fatty acids, major contributors to the development of endothelial dysfunction and atherosclerosis [2]. The data of these experimental studies are well reflected in observational studies as well as in clinical trials of patients with diabetes and atherosclerotic diseases. Today, diabetes is recognized as a major risk factor for coronary artery disease, peripheral arterial disease and cerebrovascular disease [3–5]. Even though there is plenty of data available about associations between clinical manifestations of symptomatic atherosclerotic diseases (e.g. myocardial infarction) and diabetes mellitus, there is still a lack of understanding how diabetes affects the long-term outcome in patients with subclinical atherosclerosis. Some clinical studies investigated the relationship of specific sonographic characteristics of the carotid arteries as well as the short term clinical outcome of patients with carotid atherosclerosis and diabetes [6, 7].

Similar to diabetes, atherosclerosis progresses over decades of life-time and frequently remains asymptomatic and undiagnosed up to the occurrence of a first clinical event. The role of diabetes in this context has not been sufficiently characterized. The aim of this study was to evaluate whether the degree of carotid stenosis and diabetes mellitus jointly predict long-term mortality in asymptomatic patients with carotid atherosclerosis.

## Methods

In this single-center study, we prospectively enrolled 1363 consecutive patients who underwent ultrasound investigations of the extracranial carotid arteries between March 2002 and March 2003. Study design, inclusion, and exclusion criteria have been published previously [8]. Patients with prevalent atherosclerotic carotid artery disease, defined by the presence of non-stenotic plaques or carotid stenosis of any degree that were neurologically asymptomatic at the time of screening, were enrolled. The main indications for performing ultrasound investigation were carotid bruits, prevalence of cardiovascular risk factors, and known atherosclerotic diseases in other vessel areas. Patients with current infectious, inflammatory diseases or active malignancies, symptomatic of carotid artery disease that necessitated revascularization therapy, patients having undergone bilateral carotid occlusions, bilateral stent implantation, or bilateral carotid endarterectomy, as well as patients with a myocardial infarction, stroke, coronary revascularization, or peripheral vascular surgery during the preceding 6 months, were excluded from the study. Cardiovascular and all-cause mortality were assessed by searching the national death register for the specific cause of death (according to the International Statistical Classification of Diseases and Related Health Problems, 10th Revision). Only the specific cause of death (e.g., acute myocardial infarction) was used to categorize death as either all-cause, cardiovascular, or non-cardiovascular death. In 43% of deaths, the underlying cause was assessed by autopsy.

### Clinical and laboratory data

Every enrolled patient completed a detailed study questionnaire that was reviewed by a physician assessing the patient’s medical history, current medication, biometric data, and family history. All clinical characteristics were ascertained by 2 independent observers. Antecubital venous blood samples were drawn and analyzed directly without freezing according to local laboratory standard procedures within 2 to 4 h of sampling. Serum levels of Hba1c were determined at admission (DADE Behring, IL, USA). Treating physicians and ultrasonographers were blinded for all laboratory values, color-coded duplex sonography, and grading of internal carotid artery stenosis.

### Degree of carotid stenosis

Duplex examinations at baseline were performed on an Acuson 128 XP10 with a 7.5-MHz linear array probe (Acuson, Malvern, PA). The degree of carotid artery narrowing was obtained according to 6 categories, corresponding to NASCET (North American Symptomatic Carotid Endarterectomy Trial) angiographic graduation [8, 9]. A cut-off at 50% degree of carotid narrowing, measured with ultrasound sonography, was set to obtain clinically useful measures for the effect sizes. The rationale behind this was that carotid narrowing (assessed by sonography) less than 50% is generally considered hemodynamically insignificant [9, 10]. Our interobserver agreement was adequate with respect to the absolute degree of stenosis (κ = 0.83, 95% CI 0.79 to 0.88) and with respect to progression of the disease (κ = 0.85, 95% CI 0.80 to 0.89).

### Definitions

Definitions of risk factors and comorbidities were published previously [8]. Briefly, hypertension was considered present in patients with blood pressure above 140/90 mm Hg or in patients taking antihypertensive medication. A family history of atherosclerotic disease was considered positive if its presence had been verified in a first-degree relative.

Since patients were enrolled between 2002 and 2003, diabetes mellitus was defined as fasting blood glucose levels > 125 mg/dL (i.e. > 7.0 mmol/L) according to the 1997 criteria of the American Diabetes Association [11]. In addition, according to the 2019 classification of diabetes of the American Diabetes Association, patients with baseline glycohemoglobin A1c levels ≥ 6.5% were retrospectively also defined as diabetic [12].

### Statistical methods

Continuous data are presented as median and interquartile range (range from the 25th to the 75th percentile). Discrete data are given as counts and percentages. Analysis of variance and the χ2 test were used for comparisons between, as appropriate. The log-rank test was used for comparison between groups. Event-free survival probabilities were estimated using the Kaplan–Meier method. Univariable and multivariable Cox proportional hazards models were applied to assess the association between diabetes mellitus, Hba1c, degree of carotid stenosis and the occurrence of either all-cause or cardiovascular death. The following variables were included as co-variables in every multivariable model: age (years), sex (male/female), history of myocardial infarction (binary), history of stroke (binary), peripheral arterial disease (binary), body mass index (kg/m^2^), hypertension (binary), serum creatinine (mg/dL), levels of triglycerides (mg/dL), total cholesterol levels (mg/dL), low density lipoprotein cholesterol levels (mg/dL), high sensitive C-reactive protein (mg/dL) and statin treatment (binary). The selection of the variables was defined a priori and is based on current guidelines for cardiovascular risk prediction. All of the variables listed above were included in every multivariable Cox proportional hazard model used for this study. Results of the Cox models are presented as hazard ratios (HR; 95% confidence interval [CI]). We assessed the overall model fit using Cox–Snell residuals. We also tested the proportional hazard assumption for all covariates using Schoenfeld residuals (overall test) and the scaled Schoenfeld residuals (variable-by-variable testing).

An improvement in individual risk prediction was examined using the net reclassification improvement [13]. Interactions between plasma levels of Hba1c or diabetes mellitus (binary) and degree of carotid artery stenosis were tested by entering interaction terms in the Cox proportional hazard regression models. A 2-sided *P* value of < 0.05 was considered significant. All calculations were performed with SPSS (version 20.0, SPSS Inc.) and the STATA11 software package (Stata Corp.) for Windows.

## Results

### Study population

A total of 1363 patients were enrolled in the study. Ninety-five (7%) of these patients had missing duplex ultrasound follow-up data, and 203 patients (16%) were lost to clinical follow-up, leaving 1065 patients for the final analysis. The 298 patients who had to be excluded did not significantly differ from the patients who were included in terms of baseline and demographic parameters (data not shown).

In total, 1065 patients were included in the final analysis. The median age was 69 years (IQR 61–76 years) at the time of inclusion and 668 (62.7%) were male (Table 1). Within a period of 11.9 years (IQR 6.0–12.4 years), we recorded 548 (51.5%) deaths from any cause. Of these, 367 patients (67%) died from cardiovascular causes, 142 (13.3%) of malignant diseases, and 45 (4.2%) of other causes.

**Table 1:** Baseline characteristics and risk factors of 1065 patient

Variable	Combined model				P-value
	Carotid stenosis < 50% and non-diabetic n = 486	Carotid stenosis ≥ 50% and non-diabetic n = 247	Carotid stenosis < 50% and diabetes n = 205	Carotid stenosis ≥ 50% and diabetes n = 127	
Age (years)	66.9 (59.0–75.3)	71.9 (64.1–77.5)	68.6 (61.2–75.0)	70.9 (63.5–77.7)	< 0.01
Male (binary)	290 (59.7)	158 (64.0)	138 (67.3)	82 (64.6)	0.25
History of PAD (binary)	155 (31.9)	116 (47.0)	108 (52.7)	77 (60.6)	< 0.01
History of MI (binary)	90 (18.5)	48 (19.4)	62 (30.2)	57 (44.9)	< 0.01
History of Stroke (binary)	68 (14.0)	51 (20.6)	24 (11.7)	33 (26.0)	< 0.01
Art. Hypertension (binary)	291 (59.9)	177 (71.7)	158 (77.1)	105 (82.7)	< 0.01
Current Smoker (binary)	124 (25.5)	72 (29.1)	48 (23.4)	43 (33.9)	0.14
Family history of atherosclerosis (binary)	252 (51.9)	152 (61.5)	105 (51.2)	81 (63.8)	0.01
BMI (ratio)	25.6 (23.7–28.1)	26.2 (23.7–28.3)	27.7 (30.5–24.7)	26.8 (24.2–29.6)	< 0.01
HbA1c (%)	5.7 (5.4–6)	5.9 (5.6–6.1)	7.1 (6.6–7.9)	7.1 (6.6–7.8)	< 0.01
Total cholesterol (mg/dl)	207 (178–243)	208 (181–237)	194 (169–221)	202 (170–223)	0.01
Triglycerides (mg/dl)	139 (99–201)	147 (109–205)	154 (111–235)	165 (115–237)	< 0.01
HDL cholesterol (mg/dl)	53 (43–63)	50 (42–60)	45 (39–55)	46 (38–54)	< 0.01
LDL cholesterol (mg/dl)	122 (95–151)	122 (97–146)	109 (87–137)	115 (92–138)	< 0.01
hs-CRP (mg/dl)	0.25 (0.11–0.58)	0.29 (0.13–0.68)	0.33 (0.19–0.66)	0.35 (0.15–0.79)	< 0.01
Serum creatinine (mg/dl)	1.02 (0.92–1.18)	1.07 (0.96–1.21)	1.08 (0.95–2.18)	1.13 (0.97–1.30)	0.02
Statins (binary)	247 (50.8)	161 (65.2)	126 (61.5)	86 (67.7)	< 0.01
Insulin therapy ± Oral antidiabetics (binary)	–	–	47 (22.9)	27 (21.3)	0.53*
Oral antidiabetics (binary)	–	–	101 (49.3)	65 (51.2)	0.13*

Continuous data are presented as the median and the interquartile range. Discrete data are given as counts and percentages

* P-value for the comparison between the groups “Carotid stenosis < 50% and diabetes” and “Carotid stenosis ≥ 50% and diabetes”

### Diabetes and long-term mortality

Diabetes mellitus was present in 335 (31.5%) subjects. The vast majority suffered from type 2 diabetes (95.3%). During follow up, 69.8% (169) of patients with prevalent diabetes at enrollment died compared to 46.1% (379) of patients without diabetes (Table 2). The all-cause mortality rate was 66.9% in diabetic and 44.4% in non-diabetic patients, and the cardiovascular mortality rate was 47.5% in diabetic and 28.5% in non-diabetic patients, respectively (P < 0.01). In multivariable analyses diabetes displayed a robust and independent risk for all-cause (adjusted HR 1.62, 95% CI 1.35–1.94) and cardiovascular death (adjusted HR 1.75, 95% CI 1.40–2.19). Glycohemoglobin A1c levels were significantly associated with mortality. The median HbA1c was 6.0% (IQR 5.6–6.6%) in the overall and 7.2% (IQR 6.4–8.1%) in the diabetic population and the risk of all-cause and cardiovascular mortality significantly increased in patients with elevated serum levels of Hba1c. The adjusted hazard ratio per increase of 1% of Hba1c levels was 1.21 (CI 1.12–1.32, P < 0.01) for all-cause and 1.30 (CI 1.20–1.43, P < 0.01) for cardiovascular mortality, respectively (Table 2).

**Table 2:** Results of univariable and multivariabe Cox regression analyses

Variable		All-cause mortality			Cardiovascular mortality		
		Hazard ratio	CI	P-value	Hazard ratio	CI	P-value
Univariable	n						
Diabetes mellitus	335	1.14	1.06–1.22	< 0.001	1.14	1.08–1.22	< 0.001
Per increase of 1% of hba1c	1065	1.23	1.15–1.32	< 0.001	1.30	1.20–1.41	< 0.001
Carotid stenosis ≥ 50%	374	1.67	1.40–1.97	< 0.001	1.70	1.39–2.08	< 0.001
Combined model							
Carotid stenosis < 50%; Ø DM^a^	522	Ref					
Carotid stenosis ≥ 50%; Ø DM	276	1.65	1.33–2.06	< 0.001	1.71	1.30–2.25	< 0.001
Carotid stenosis < 50%; DM	166	1.86	1.48–2.34	< 0.001	2.13	1.61–2.81	< 0.001
Carotid stenosis ≥ 50%; DM	101	3.23	2.53–4.13	< 0.001	3.67	2.73–4.94	< 0.001
Multivariable							
Diabetes mellitus	335	1.14	1.06–1.22	< 0.001	1.14	1.08–1.22	< 0.001
Per increase of 1% of hba1c	1065	1.21	1.12–1.32	< 0.001	1.30	1.20–1.43	< 0.001
Carotid stenosis ≥ 50%	374	1.28	1.30	0.004	1.28	1.03–1.60	0.025
Combined model							
Carotid stenosis < 50%; Ø DM^a^	522	Ref					
Carotid stenosis ≥ 50%; Ø DM	276	1.27	1.01–1.59	0.040	1.26	0.96–1.64	0.072
Carotid stenosis < 50%; DM	166	1.56	1.22–1.99	< 0.001	1.78	1.33–2.39	< 0.001
Carotid stenosis ≥ 50%; DM	101	2.22	1.71–2.99	< 0.001	2.35	1.71–3.24	< 0.001

*CI* confidence interval, *HR* hazard ratio, *hsCRP* high sensitivity C-reactive protein

^a^Reference category; adjusted for age, sex, body mass index, hypertension smoking, history of peripheral artery disease, history of stroke history of myocardial infarction, low-density lipoprotein cholesterol levels, triglyceride levels, statin treatment, serum creatinine, hsCRP

### Diabetes and degree of carotid stenosis

377 (35.4%) patients had unilateral or bilateral carotid artery narrowing of ≥ 50% at enrollment. To assess the joint effect of diabetes and carotid stenosis on long-term outcome, the patient population was stratified into 4 groups according to the degree of carotid narrowing and the frequency of diabetes. Group 1 were non-diabetic patients with carotid narrowing < 50%. Group 2 included diabetic patients with carotid narrowing < 50%. Group 3 represented patients with carotid narrowing ≥ 50% but without diabetes, and group 4 patients with both carotid stenosis ≥ 50% and diabetes.

**Fig. 1:**
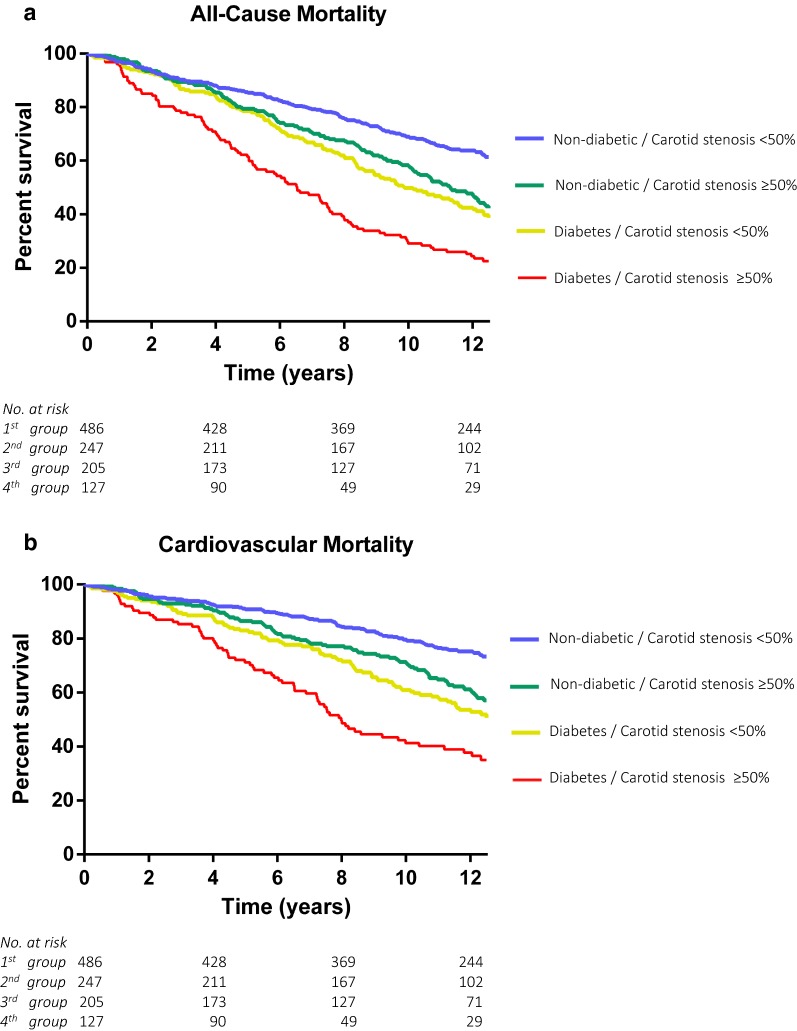
Kaplan–Meier estimates of all-cause and cardiovascular mortality. During a median follow-up time of 11.81 years (IQR, 6.01–12.43) according to degree of carotid stenosis and prevalence of diabetes mellitus. Group 1 was defined by carotid narrowing of < 50% and without diabetes. Group 2 included patients with carotid narrowing ≥ 50% and without diabetes. Group 3 represented patients with carotid narrowing < 50% and diabetes, and group 4 patients with both carotid stenosis ≥ 50% and diabetes. Log-rank test was used for the overall comparison among groups. **a** Kaplan–Meier estimates of all-cause mortality. 549 (51.5%) all-cause deaths were recorded. **b** Kaplan–Meier estimates of cardiovascular mortality. 367 (34%) cardiovascular deaths were recorded

The cumulative 12-year survival rates in groups 1 to 4 were 62%, 43%, 40%, and 21% for all-cause death and 76%, 63%, 57%, and 44% for cardiovascular death (P < 0.01; Fig. 1) Adjusted HRs for the risk of all-cause death in groups 2 to 4 were 1.27 (CI 1.01–1.59), 1.56 (CI 1.22– 1.99), and 2.22 (CI 1.71–2.99; P < 0.01), and for cardiovascular death 1.26 (CI 0.96–1.64), 1.63 (CI 1.20–1.2.21), and 2.40 (CI 1.72–3.34; P < 0.01), compared with the first group (Table 2).

Improvement in individual risk stratification with combined assessment of degree of carotid stenosis and diabetes mellitus was confirmed by a significant improvement in the net reclassification improvement, with 39% (± 6%; P < 0.001) for all-cause and 37% (± 6%; P < 0.001) for cardiovascular mortality, respectively, as compared with degree of carotid stenosis alone. We did not observe any significant interactions between diabetes or Hba1c, degree of carotid artery stenosis and mortality (P-value for interaction with diabetes = 0.64, and with Hba1c ≥ 50% = 0.39).

## Discussion

Our study found a clear association between the presence of carotid atherosclerosis and mortality among diabetic patients. In a combined assessment, we identified that subjects with diabetes and asymptomatic carotid stenosis ≥ 50% are at an exceptional high risk for adverse outcome. If a patient at time of inclusion was diagnosed with both diabetes mellitus and carotid stenosis above 50%, the patient had only a ~ 20% chance to survive the following 12 years. These findings are more than alarming, considering that the whole cohort had a survival rate of ~ 50%. Further, diabetic patients with carotid stenosis ≥ 50% had a near 2.5-fold increase in risk of cardiovascular death compared to non-diabetic patients with carotid atherosclerosis below 50% narrowing. These latter findings were independent of various established cardiovascular risk factors or previous cerebro- or cardiovascular events. We further identified that combining carotid stenosis with diabetes mellitus improved the risk stratification by near 40% for the risk of all-cause and cardiovascular death, respectively. In contrast, the clinical diagnosis diabetes mellitus, based on hba1c and serum glucose levels alone, displayed a highly significant, but rather weak association with (cardiovascular) mortality in patients in asymptomatic carotid atherosclerosis after 12-years of follow-up.

### Diabetes, atherogenesis and cardiovascular outcome

The association between diabetes and the development of cardiovascular disease has been well established in the last decades or so [3–5]. More recently, it has been shown that patients with late onset autoimmune diabetes in the adult (LADA) are at increased macrovascular risk despite a “healthier” risk profile as compared to patients with type 2 diabetes [14]. It appears, that especially in “younger” populations inflammation and plaques composition are main triggers for adverse outcome, since atherosclerotic plaques with thin fibrous caps and large necrotic cores are clearly associated with cardiovascular events and death [15]. In this context, fibrinogen was recently found to be inversely associated with intraplaque hemorrhage- and necrotic core-volume, independently of inflammation [16]. Fernández-Friera et al. lately described that arterial inflammation is highly prevalent in middle-aged individuals with known subclinical atherosclerosis, and thus may trigger early cardiovascular events [17].

Our results are in line with previous outcome studies which predominantly investigated the role of carotid intima media thickness in patients at cardiovascular risk [18–22]. There have also been reports that information about the presence of carotid atherosclerosis can be used for the prediction of outcome in patient with coronary artery disease [23] and that the combined assessment of IMT and interadventitia common carotid artery diameter is useful to stratify a patient’s individual cardiovascular risk profile [24]. However, our study oversees a 12-year period of follow-up and provides robust data on long term outcome in patients with prevalent or advanced carotid atherosclerosis among a well-defined cohort.

Diabetes as well as carotid atherosclerosis are frequently referred to as “silent killers” and our study not only confirms, but also emphasizes the strong additive effect of both risk factors. The prevalence of carotid atherosclerosis in patients older than 60 years is high and the same holds true for diabetes [25, 26]. Based on our data, we believe that a screening method for subclinical atherosclerosis in patients with diabetes might be a useful tool for risk stratification. According to the current guidelines of the American Diabetes Association for Cardiovascular Disease and Risk Management, screening for coronary artery disease in asymptomatic patients with diabetes mellitus and a high risk profile for cardiovascular disease, is currently not recommended [27]. This is mainly attributed to the screening methods for coronary artery disease which are costly, often risky for the patient, time-consuming and require certain infrastructure. On the contrary, the performance of an ultrasound investigation of the carotid arteries is simple, cost-effective and does not pose any risk to the patient.

### Anti-diabetic medication and cardiovascular outcome

Various novel anti-diabetic drugs such as Sodium-Glucose Co-Transporter 2 (SGLT2) inhibitors or dipeptidyl-peptidase 4 (DPP-4) inhibitors have recently been introduced to the market and some of them have the capacity to significantly reduce cardiovascular events and even cardiovascular mortality [28, 29]. The findings sparked a debate in the community whether the very pronounced improvement of cardiovascular outcome can be attributed solely to the anti-diabetic effects of these drugs. Consequently, numerous post-trial studies are now evaluating the cardiovascular protective properties of these drugs [30]. In this context, it had been recently demonstrated that sitagliptin improves tissue characteristics of the carotid arterial wall [31].

Our data suggest that sonographic assessment of the degree of carotid stenosis at a single time-point can easily stratify diabetic patients in high and low risk groups for adverse cardiovascular outcome. Since the identification of high risk patients is of major importance for these trials, we believe that future drug related research could benefit from our findings.

### Limitations

Although our results suggest a strong association between diabetes and outcome in patients with subclinical carotid atherosclerosis, we are aware of some limitations to our study. The study was initially designed to evaluate inflammatory biomarkers and we therefore have a lack of information about the dosage of insulin and the specific type of oral anti-diabetic drugs as well as baseline fasting plasma glucose levels. In addition, time-dependent factors, change of the therapeutic regimen and socio or environmental factors may influence the relationship between carotid stenosis, diabetes and long-term mortality. Due to advances in ultrasound technology, imaging has improved the assessment of plaques composition [32]. Since baseline investigations were performed in the early 2000s, we cannot provide data of the specific carotid plaque composition.

## Conclusion

Diabetic patients with carotid stenosis ≥ 50% are at exceptional high risk for all-cause and cardiovascular death. Thus, routinely ultrasound investigation of the carotid arteries might be a valuable prognostic tool for patients with diabetes mellitus. However, further research is warranted to evaluate the clinical usefulness of ultrasound investigations of the carotid arteries as a screening tool in patients with diabetes mellitus and whether adjusted therapeutically intervention improves outcome in these patients.

## Data Availability

Patient data cannot be shared, because of protection of data privacy.

## Abbreviations

AGEs: advanced glycation end products
DPPI: dipeptidyl-peptidase 4 inhibitors
HbA1c: glycohemoglobin A1c
HR: hazard ratio
IQR: interquartile range
SGLT2: sodium-glucose co-transporter 2 inhibitors
